# Carbon Nanotubes Improved the Germination and Vigor of Plant Species from Peatland Ecosystem Via Remodeling the Membrane Lipidome

**DOI:** 10.3390/nano10091852

**Published:** 2020-09-16

**Authors:** Md. Hossen Ali, Jean-Marie Sobze, Thu Huong Pham, Muhammad Nadeem, Chen Liu, Lakshman Galagedara, Mumtaz Cheema, Raymond Thomas

**Affiliations:** 1 School of Science and the Environment/Boreal Ecosystem Research Facility, Grenfell Campus, Memorial University of Newfoundland, Corner Brook, NL A2H 5G5, Canada; mhali@grenfell.mun.ca (M.H.A.); tpham@grenfell.mun.ca (T.H.P.); cliu@grenfell.mun.ca (C.L.); lgalagedara@grenfell.mun.ca (L.G.); mcheema@grenfell.mun.ca (M.C.); 2 Northern Alberta Institute of Technology, Boreal Research Institute, 8102-99 Avenue, Peace River, AB T8S 1R2, Canada; jeanmars@nait.ca

**Keywords:** carbon nanoparticles, carbon nanotubes (CNT), germination rate, membrane lipid, seed dormancy, seedling vigor

## Abstract

Application of the nanopriming technique to alleviate seed dormancy has shown promising results in various agricultural crop species. However, there is a dearth of knowledge regarding its potential use in native peatland boreal forest species to alleviate seed dormancy and improve their propagation or vigor for forest reclamation activities. Herein, we demonstrate the use of nanopriming with carbon nanotubes (CNT) to alleviate seed dormancy, improved seed germination, and seedling vigor in two boreal peatland species. Bog birch (*Betula pumila* L.) and Labrador tea (*Rhododendron groenlandicum* L.) seeds with embryo or seed coat dormancy were nanoprimed with either 20 or 40 µg/mL CNT, cold stratified at 2–4 °C for 15 days, and allowed to germinate at room temperature. The emerged seedlings’ lipidome was assessed to decipher the role of lipid metabolism in alleviating seed dormancy. We observed significant (*p* < 0.05) improvement in seedling germination and seedling vigor in seeds primed with multiwalled carbon nanotubes functionalized with carboxylic acids. Phosphatidylcholine (PC 18:1/18:3), phosphatidylglycerol (PG 16:1/18:3), and lysophosphatidylcholine (LPC 18:3) molecular species (C18:3 enriched) were observed to be highly correlated with the increased seed germination percentages and the enhanced seedling vigor. Mechanistically, it appears that carbon nanoprimed seeds following stratification are effective in mediating seed dormancy by remodeling the seed membrane lipids (C18:3 enriched PC, PG, and LPC) in both peatland boreal forest species. The study results demonstrate that nanopriming may provide a solution to resolve seed dormancy issues by enhancing seed germination, propagation, and seedling vigor in non-resource boreal forest species ideally suited for forest reclamation following anthropogenic disturbances.

## 1. Introduction

Seed dormancy refers to situations whereby seeds are incapable of germinating under conditions that are ideally favorable to support germination [1,2,3]. This can be due to deficiency or excesses of water, light, temperature, gasses, presence of mechanical restrictions affecting seed coats, and unsuitable growth hormone levels [4]. Seed dormancy can be physical, morphological, or physiological in nature [5,6]. For example, physical dormancy is characterized by seed coat impermeability to water and gases, which limits water uptake and gas exchange in the seed [7]. This form of dormancy can only be resolved by softening or cracking the seed coat through physical, biological, and/or chemical scarification techniques [5,6,8]. Conversely, morphological dormancy is observed in seeds with an immature embryo [8], while, in physiological dormancy, the seeds contain chemical growth regulators that inhibit seed germination [9].

Bog birch (*Betula pumila* L.) is a deciduous shrub native to peatland ecosystems in boreal forests across North America [5,10]. The seeds of bog birch are 1–3 mm long and have hard seed coats possessing embryo dormancy and results in delayed and poor germination [11]. Conversely, the seeds of Labrador tea (*Rhododendron groenlandicum* L.) are very tiny and germinate to approximately 80%, but seedling vigor is low [5]. Both bog birch and Labrador tea are important indicator species in the boreal peatland ecosystem and tend to be among the first affected during anthropogenic disturbances such as resource mining [5,10]. Mass propagating of these species is important for their redeployment during boreal forest reclamation or restoration activities [5].

Seed germination represents the most common mass propagation technique, and successful seedling establishment on the disturbed sites in peatland ecosystem depends on many factors including the ability to withstand varying changes in environmental conditions, which is a function of seedling vigor [12]. Seed vigor is defined as the ability of seeds to germinate and become established across a range of sites with varying environmental condition [2]. As such, seedling vigor is an important indicator used to assess seedling quality and emergence as well as propensity to be established across a range of field and environmental conditions [5]. Furthermore, the electrical conductivity (EC) of seeds is a widely used technique to assess the seed vigor [13], and is shown to be highly associated or correlated with seedling emergence [14]. Electrolytes leaked from the cell membrane of poor vigor seedlings increase EC readings. As such, high levels of membrane leakage or EC values are characteristic or indicative of poor seedling vigor and emergence under field conditions [15].

Seed priming has been used as a successful technique to break dormancy, improve seed germination, and seedling vigor in many agriculture crops [5,6,16,17,18]. Priming also ensures uniformity in seed germination, growth, and improvement in germination rate, while also enhancing seedling vigor by inducing inherent stress tolerance in the germinating seeds. Several priming techniques have been used to improve seed germination in many agricultural crop species and include halopriming (salt solution), biopriming (solution of valuable organisms), osmopriming (osmotic solution), hormonal priming (solutions of plant hormones), magneto-priming (nearness of a magnetic field), matriconditioning (solutions blend with a strong transporter), and nanopriming (hydrate with nanoparticle solution) [5,6,19]. Several recent studies have shown nanopriming to be very effective in improving seed germination in agricultural crops [16]. The increased germination was observed to occur concomitant with enhanced seedling vigor, root, and stem growth in conditions that were not ideal for plant growth [20]. Given the success of nanopriming in improving germination in agricultural species, the potential exists that this success could be replicated in native boreal forest plant species with seed dormancy issues, low seed germination, and seedling vigor. In our previous study, we demonstrated that nanopriming enhanced the seed germination and seedling vigor in the upland boreal forest species [5,6].

In many plants during germination, seed lipids are hydrolyzed into fatty acids and glycerol by lipases [5]. These fatty acids are further converted into acetyl-CoA by the process of β-oxidation to make cellular ATP to drive various biological processes in the cell during plant growth. The membrane of seeds is usually reorganized naturally during germination, where an increase in the level of plastidic lipids such as lysophosphatidic acid (LPA), phosphatidic acid (PA), diglycerides (DG), phosphatidylglycerol (PG), phosphatidylinositol (PI), phosphatidylserine (PS), phosphatidylethanolamine (PE), and monogalactosyldiacylglycerol (MGDG) have been reported [6,21]. For example, phosphatidic acid (PA) was shown to be remodeled during germination in soybean seeds, where the level of PA was observed to decrease at the beginning of germination before increasing exponentially during phase I and II, followed by a decline at the end of germination [22]. Several studies have also shown strong correlations between seed lipid metabolism and germination [5,6]. For instance, the levels of complex seed membrane lipids such as phospholipids, galactolipids, and sphingolipids were observed to increase several folds during germination in the seeds of *Medicago sativa* [21]. Specifically, the increase in phospholipids, particularly phosphatidylcholine (PC), appeared higher than all other seed membrane lipid classes during germination. These findings demonstrate that altered seed membrane lipid metabolism plays an essential role in seed germination [6,23]. How, this is related to successfully resolving seed dormancy, low germination, and improving seedling vigor is unclear. Furthermore, nanopriming has been applied to resolve seed dormancy and low germination in several agricultural crop species, but this has not been applied previously to boreal forest species distributed in peatland ecosystems with seed dormancy, low germination, or seedling vigor issues except in our previous study [6]. We demonstrated the effects of nanopriming on lipid remodeling in upland boreal forest species, however, there is a scarcity of literature regarding the effect of nanopriming on membrane lipid metabolism and the resolution of seed dormancy or low seedling vigor in peatland species [5,6].

Thus, we hypothesize that nanopriming of the dormant peatland boreal forest species seeds with selected carbon nanotubes (CNTs) will improve seed germination and seed vigor via remodulation of the seed or seedling membrane lipidome. This study was performed to evaluate whether nanopriming with selected CNTs is effective at improving seed germination, seedling growth, and seed vigor in two peatland boreal forest species with seed dormancy and germination issues. The outputs of this research could be applied in forest regeneration or reclamation efforts by incorporating CNTs into propagation techniques.

## 2. Materials and Methods

### 2.1. Plant Material and Experimental Treatments

Seeds from two peatland boreal forest species, bog birch and Labrador tea, were obtained from the Northern Alberta Institute of Technology (NAIT) Boreal Research Institute as explained in previous work [5]. All seeds were stored at −20 °C prior to experimentation. The study was designed based on four experimental treatments: (*i*) multiwall carbon nanotubes (MWCNTs); (*ii*) MWCNT functionalized with carboxylic acids (MWCNT–COOH); (*iii*) graphene; and (*iv*) deionized water (DI) as control.

Twenty-five seeds (25) from each species were primed overnight in glass vials containing 8 mL solutions of either 20 µg/mL or 40 µg/mL of CNTs dissolved in DI water [5]. CNTs were thoroughly dissolved in DI water following sonication for 15 min (Qsonica Q700, Model: CL-334, Boston Laboratory Equipment, Newtown, CT, USA). The treatments were replicated three times, with one set of seeds primed with and without CNTs cold stratified, and a corresponding set of seeds excluded from cold stratification (incubated at room temperature). The primed seeds were germinated in condensation free Petri plates (P5481, Sigma-Aldrich) padded with wet germination grade Whatman filter paper (CA28297-216, VWR). Seeds were incubated for three weeks at room temperature (25 °C) and illuminated with light emitting diode (LED) lights 24 h per day (2100 lumens). Water (1 mL) was added periodically to the filter paper during germination to keep the seeds hydrated [5]. For cold stratification, seeds were primed using the same conditions as above, except that seeds were incubated at a temperature of 2—4 °C for 15 days, after which the seeds were then exposed to germination conditions at room temperature (25 °C) under continuous LED lighting. The treatments are summarized as follows:

CNT: Imbibition for 24 h with select CNTs before exposure at room temperature.
CNT + stratification: Imbibition for 24 h with select CNTs and incubated in the fridge at 2—4 °C for 15 days before exposure at room temperature.

### 2.2. Tetrazolium Test

A tetrazolium test was conducted to evaluate seed viability and explained in detail in our previous work [6]. Briefly, three biological replications consisting of a total 20 seeds of each species were imbibed in DI water under two conditions (with and without each nanotubes for six h at 25 °C). A scalpel blade was then used to cut open the seeds through their embryo. Half of each seed was then immersed in tetrazolium solution (2,3,5-triphenyl tetrazolium chloride at a concentration of 0.050% (*w*/*v*)) for about 6 h [24]. The excess stain from the seeds was rinsed using DI water and the structure of the stained seeds was evaluated using a low power (0.5×) light microscope (Nikon SMZ1500, Valley Microscope, Nikon, Japan) to assess the seed viability. Viable seeds were stained bright red, while non-viable or dead seeds were unstained or stained white or light red. These evaluations led to three classification of seeds as follows: totally stained, unstained, and partially stained [25].

### 2.3. Electrical Conductivity

Electrical conductivity (EC) test was done using a handheld EC meter (Field Scout EC Meter, Spectrum Technologies, Inc., Aurora, IL, USA) to assess membrane integrity and seed vigor. Three sets of twenty-five seeds from each treatment were soaked in CNPs (MWCNT–COOH, MWCNT, graphene) or DI water overnight and the conductivity recorded. Low EC readings were indicative of superior seed vigor [14].

### 2.4. Evaluation of Abnormal and Normal Seedlings

The normal and abnormal seedlings following germination were assessed through standard procedures and explained in detail in our previous work [5,6].

### 2.5. Germination Evaluation

The germination rate and seed vigor were measured by observing germination, shoot, and root growth over the entire period of the experiment [5]. The number of emerged seeds and the total seeds at each point in time were counted daily to track the germination rate in each treatment [5]. The analysis was done by measuring the shoot and root length of each seedling. After measurement, seedlings were stored at −80 °C for lipid analysis. The seed germination rate in each treatment was determined by the following equation:

(1) $$Germination\;\left(\%\right)=\frac{number\;of\;germinated\;seeds}{Number\;of\;total\;seeds}\times 100$$

### 2.6. Seedling Vigor Index (SVI)

The seedling vigor index (SVI) is an important parameter of seed quality and used to assess germination enhancement and viability of a seed lot under varying environmental conditions [5,6]. Three days after germination, the seedling length (cm) was measured using a ruler and used to calculate the SVI as follows:

(2) $$SVI=\frac{Seedling\;length\;\left(cm\right)\;}{100}\times \%\;germination$$

### 2.7. Lipid Extraction

For lipid extractions, germinated seedlings (three days old with seed still attached) in each treatment replicate were collected and incubated in hot isopropanol for 15 min as explained in detail in our previous work [6]. Briefly, the lipids were extracted using a modified version of the Bligh and Dyer method [26]. Ten mg of seedlings were weighed in glass centrifuge tubes. Afterward, 1 mL of methanol containing 0.01% butylated hydroxytoluene (BHT), 1 mL of chloroform, and 0.8 mL of water were added to the sample. The sample was homogenized using a probe blender (Heraeus Megafuge16, Thermo Scientific). The sample mixture was centrifuged at 5000 rpm for 15 min. After centrifugation, the organic layer (bottom), which contained the lipids, were removed and added to pre-weighed 4 mL sample vials containing polytetrafluoroethylene (PTFE) lined caps (VWR, Mississauga, ON, Canada). The organic phase of the sample was then dried under nitrogen and vials with the sample re-weighed to establish the amount of lipids extracted [26]. The extracted lipids were re-suspended in 1 mL chloroform:methanol (1:1 *v*/*v*) and kept at −20 °C for lipid analysis.

### 2.8. Analysis of Plant Membrane Lipids

Ultra-high-performance liquid chromatograph coupled to C30 reverse phase chromatography and heated electrospray ionization high-resolution tandem mass spectrometry (UHPLC-C30RP-HESI- HRAMS/MS-MS) was employed to evaluate the plant membrane lipid classes and their associated molecular species [5,6]. A Thermo Scientific Q-Exactive Orbitrap mass spectrometer (Thermo Scientific, Berkeley, MO, USA) coupled to an automated Dionex UltiMate 3000 UHPLC system operated by the Chromeleon software was used to analyze the lipids from the seedlings. A Thermo Fisher Scientific (Mississauga, ON, Canada) Accucore C30 column (150 mm × 2 mm I.D., particle size: 2.6 µm, pore diameter: 150 Å) was employed to separate the lipids. The solvent system used to separate the lipid mixture on the C30 column was as follows: (i) Solvent A consisted of acetonitrile: H_2_O (60:40 *v*/*v*) containing 10 mM ammonium formate and 0.1% formic acid and (ii) Solvent B consisted of isopropanol:acetonitrile:water (90:10:1 *v*/*v*/*v*) with 10 mM ammonium formate and 0.1% formic acid.

UHPLC-C30RP separation was carried out at 30 °C (column oven temperature) with a flow rate of 0.2 mL/min. Aliquots (10 µL) of the lipid mixture suspended in chloroform: methanol (2:1 *v*/*v*) was injected into the machine. The following gradient system was used for separating the lipid classes and molecular species: solvent B increased to 30% in 3 min; to 43% in 5 min, to 50% in 1 min, to 90% in 9 min, to 99% in 8 min, and finally maintained at 99% for 4 min. The column was re-equilibrated to starting conditions (70% solvent A) for 5 min before each new injection. Full scans (HESI-MS) and MS/MS acquisitions were performed on the mass spectrometer in negative and positive modes, and the instrument was controlled by X-Calibur software 4.0. The following parameters were used to operate the Orbitrap mass spectrometer: sheath gas: 40, auxiliary gas: 2, ion spray voltage: 3.2 kV, capillary temperature: 300 °C; S-lens RF: 30 V; mass range: 200–2000 m/z; full scan mode at a resolution of 70,000 m/z; top-20 data-dependent MS/MS at a resolution of 35,000 m/z and collision energy of 35 (arbitrary unit); injection time of 35 min for C30RP chromatography; isolation window: 1 m/z; automatic gain control target: 1e5 with dynamic exclusion setting of 5.0 s. The instrument was externally calibrated to 1 ppm using electrospray ionization (ESI) negative and positive calibration solutions (Thermo Scientific, Waltham, MO, USA). Tune parameters were optimized using PC 18:1(9Z)/18:1(9Z), Cer d18:1/18:1(9Z), PG 18:1(9Z)/18:1(9Z), sulfoquinovosyl diacylglycerols [SQDG] 18:3(9Z,12Z,15Z)/16:0, monogalactosyl diglyceride [MGDG] 18:3(9Z,12Z,15Z)/16:3(7Z,10Z,13Z), and digalactosyldiacylglycerol [DGDG] 18:3(9Z,12Z,15Z)/18:3(9Z,12Z,15Z) lipid standards (Avanti Polar Lipids, Alabaster, AL, USA) in both negative and positive ion modes. The X-Calibur 4.0 Thermo Scientific, Waltham, MO, USA) and LipidSearch version 4.1 (Mitsui Knowledge Industry, Tokyo, Japan) software packages were used to process all MS data. LipidSearch 4.1 was employed to identify and quantify the lipid classes and lipid molecular species in the experiment [5,6].

### 2.9. Data Analysis

Statistical analysis was performed using XLSTAT (Premium version, Addinsoft, New York, NY, USA). Analysis of variance (ANOVA) was used to determine treatment effects. Fisher’s least significant difference (LSD) test at α = 0.05 was used as post-hoc for mean comparisons, when treatments were significantly different from each other. Redundancy analysis (RDA) and Pearson correlation coefficient tests were employed to determine associations between variables. All charts and graphs were created using Sigma Plot software (Sigma Plot 12.5, Systat Software Inc., San Jose, CA, USA).

## 3. Results

### 3.1. Tetrazolium Test

**Figure 1:**
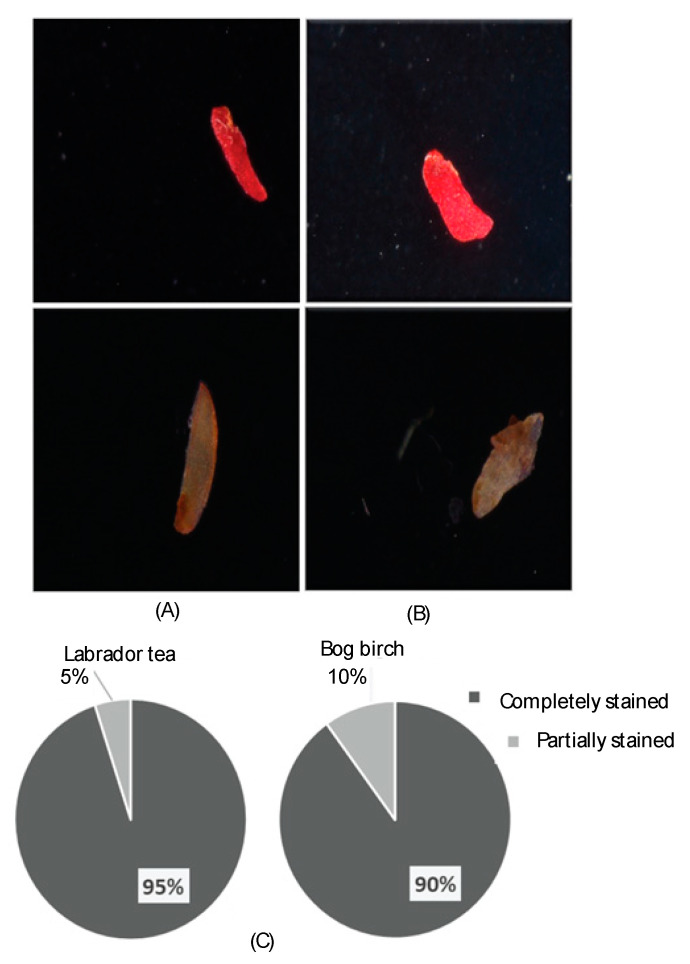
Tetrazolium test results showing viability of seeds following treatments. (**A**) Viable seed of Labrador tea were stained bright red and non-viable seed are white/light red. (**B**) Viable seeds of bog birch stained bright red and non-viable seeds are white/light red. (**C**) Percentages of completely stained and partially stained seeds.

The tetrazolium test was used to evaluate seed viability. Viable seeds were stained a bright red color, while the dead seeds were stained dark red, pink, or not at all (Figure 1). For both bog birch and Labrador tea species, 90 and 95% seeds were observed as viable seeds, respectively. In contrast, 10 and 5% of seeds were partially stained for bog birch and Labrador tea, respectively (Figure 1).

### 3.2. Electrical Conductivity Test

**Figure 2:**
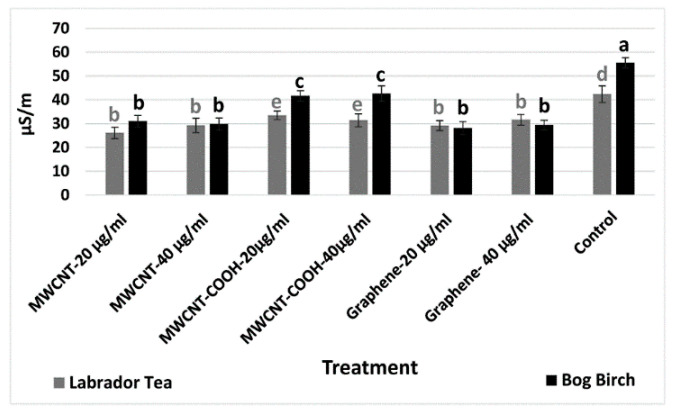
Electrical conductivity measures of both Labrador tea and bog birch. Values in the bar chart (µS/m) represent the means  ±  standard errors. Means that were significantly different (α = 0.05) are denoted by different letters. Control = no CNTs added. MWCNT–COOH = multiwall carbon nanotubes functionalized with carboxylic acid, MWCNT = multiwall carbon nanotubes.

Low EC measures indicate higher seed vigor following nanopriming. For bog birch species, the EC was 55 µS cm^−1^ in the control, which was significantly higher than that of all the treatments (Figure 2). In all treatments, the EC ranged from 20 to 35 µS cm^−1^. These results indicate that seeds primed in the presence of CNTs had improved seed vigor compared to those hydro primed without nanoparticles. In the case of the Labrador tea species, the EC was 40 µS cm^−1^ in the control, which was significantly higher than all the nanoprimed treatments, with EC values ranging from 25 to 30 µS cm^−1^ (Figure 2).

### 3.3. Evaluation of Normal and Abnormal Seedlings

**Figure 3:**
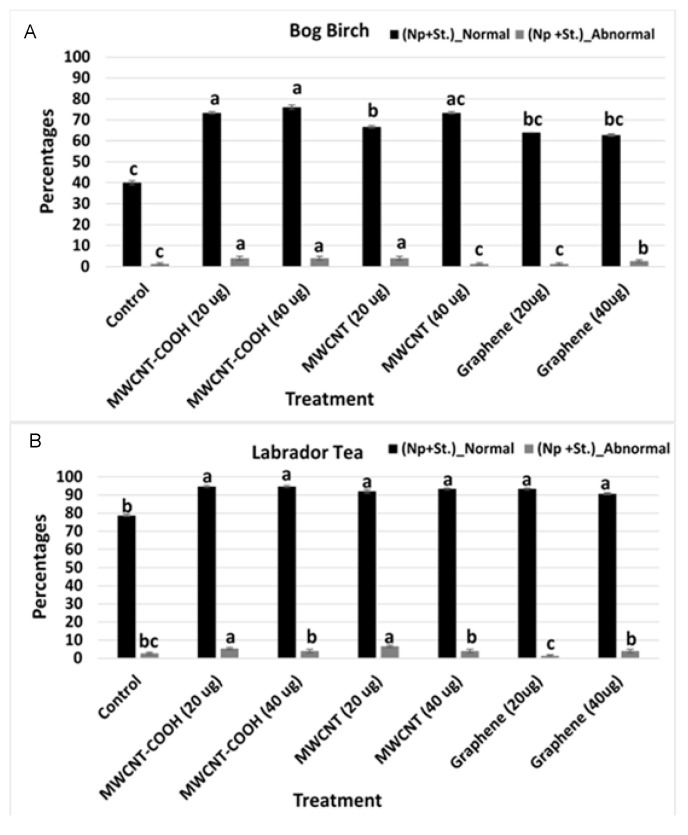
Percentages of normal and abnormal seedlings for all treatments and the control for bog birch (**A**) and Labrador tea (**B**). Values in the bar chart represent the means  ±  standard errors. Means that were significantly different (α = 0.05) are denoted by different letters. Control = no CNTs added. MWCNT–COOH = multiwall carbon nanotubes functionalized with carboxylic acid, MWCNTs = multiwall carbon nanotubes. Np + St = nanoprimed and stratified. Normal = normal seedlings, abnormal = abnormal seedlings.

The control seeds primed in DI water and stratified produced 40% normal seedlings. This was significantly lower than seeds nanoprimed with different concentrations of CNTs (Figure 3A). Between 65 to 75% of the nanoprimed seedlings were normal. The highest percentage of normal seedlings were observed in seeds nanoprimed with MWCNT–COOH (20 and 40 µg/mL). For Labrador tea species, a similar trend was observed where the lowest percent normal seedlings was observed in the control (hydroprimed) and stratified. All of the nanoprimed treatments had significantly higher normal seedlings. This ranged from 90 to 98% compared to 80% in the control treated seeds (Figure 3B). When seeds were primed, but not stratified, the germination percent was very low regardless of the treatment (Figure 3A,B).

### 3.4. Germination Percentages

**Figure 4:**
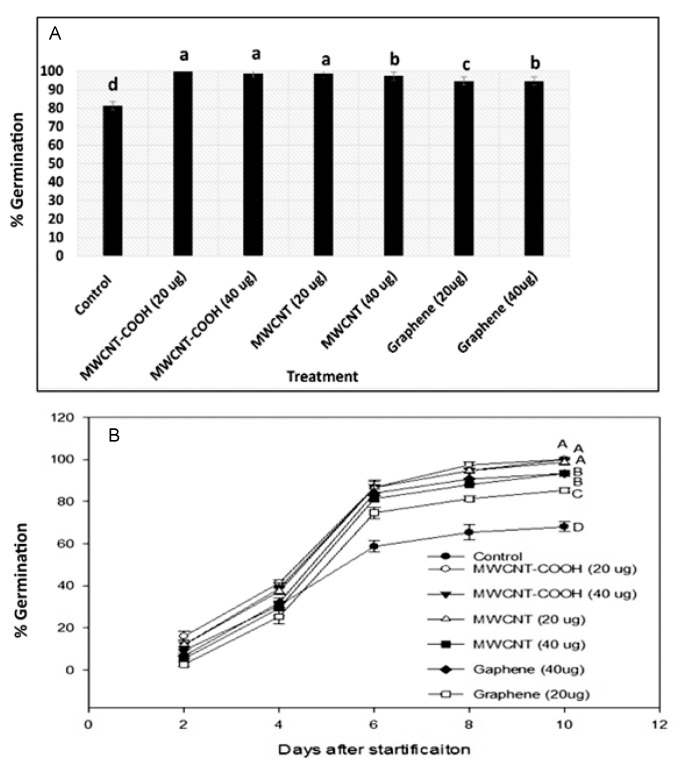
Effects of cold stratification and nanopriming on the total seed germination (**A**) and germination rate of Labrador tea (**B**). Values in bar chart represent the means ± standard errors. Means that were significantly different (α = 0.05) are denoted by different letters. Control = no CNTs added. MWCNT–COOH = multiwall carbon nanotubes functionalized with carboxylic acid, MWCNTs = multiwall carbon nanotubes.

CNT primed and stratified Labrador tea seeds had very high seed germination in all treatments compared to the control. We observed for all treatments that the germination was between 95–100%, while it was about 80% in the control. The highest percent germination (100%) was observed in MWCNT–COOH primed and stratified seeds at 20 µg (Figure 4A).

**Figure 5:**
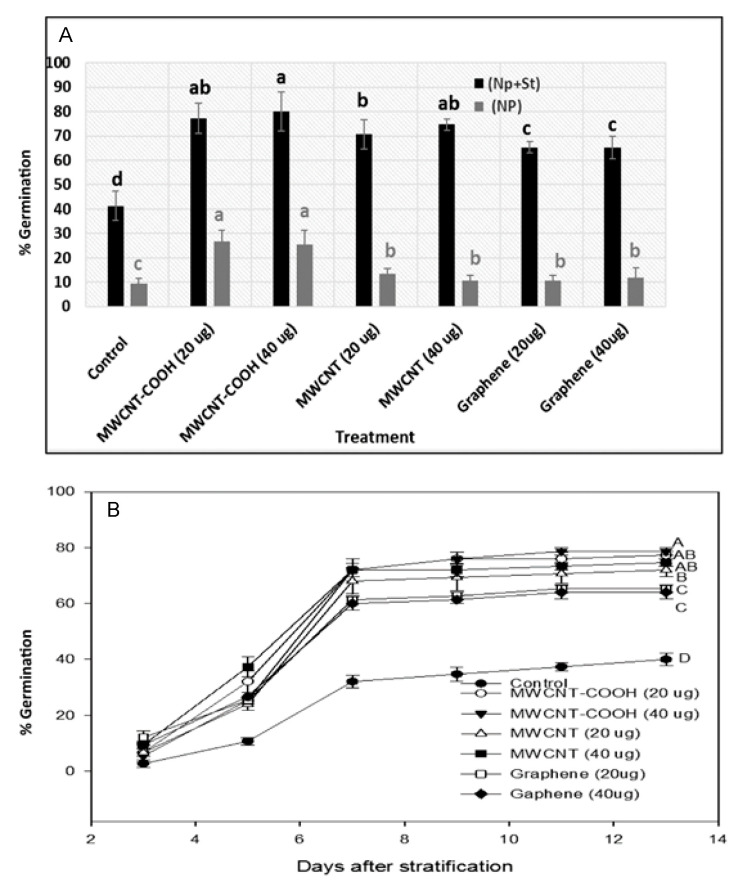
Effects of cold stratification and nanopriming on total seed germination (**A**) and germination rate (**B**) of bog birch. Values in bar chart represent the means ± standard errors. Means that were significantly different (α = 0.05) are denoted by different letters. Control = no CNTs added. MWCNT–COOH = multiwall carbon nanotubes functionalized with carboxylic acid, MWCNTs = multiwall carbon nanotubes.

The rate of germination in Labrador tea was highest in both concentrations of MWCNT–COOH (20 µg/mL and 40 µg/mL) and MWCNTs (20 µg/mL). Germination began at two days after stratification and stopped at 10 days after stratification in all treatments (Figure 4). Overall, the seeds treated with MWCNT–COOH (20 and 40 µg/mL) had the fastest and most uniform germination over time, and the germination was 100% compared to only 70% in the control (Figure 4B). For bog birch, first germination took place at three days after stratification and germination stopped at 13 days after stratification. Among all treatments, the germination was highest in the MWCNT–COOH (40 µg/mL) treatment (80%) and was lowest in the control (40%) (Figure 5B).

Seeds nanoprimed with MWCNTs functionalized with carboxylic acids (MWCNT–COOH) at both 20 and 40 µg/mL had higher germination and germination rates compared to the control treatment. Up to a 25% increase in germination was observed in bog birch seeds primed with solutions of MWCNT–COOH. For MWCNTs (20 µg/mL and 40 µg/mL) and graphene (20 µg/mL and 40 µg/mL), the germination percentages ranged from 10 to 12% whereas it was less than 10% in the control. All treatments were significantly higher than the control (Figure 5B). Bog birch seeds nanoprimed and stratified had significantly higher germination compared to all other treatments. Among all the nanoprimed and stratified seed treatments, MWCNT–COOH (20 µg/mL and 40 µg/mL) showed the highest germination of 80%. In the case of MWCNTs (20 µg/mL and 40 µg/mL), the percent of seeds germinated was about 70%, while it was about 65% for graphene (20 µg/mL and 40 µg/mL) compared to 40% in the control (Figure 5).

### 3.5. Seedling Vigor Index (SVI)

**Figure 6:**
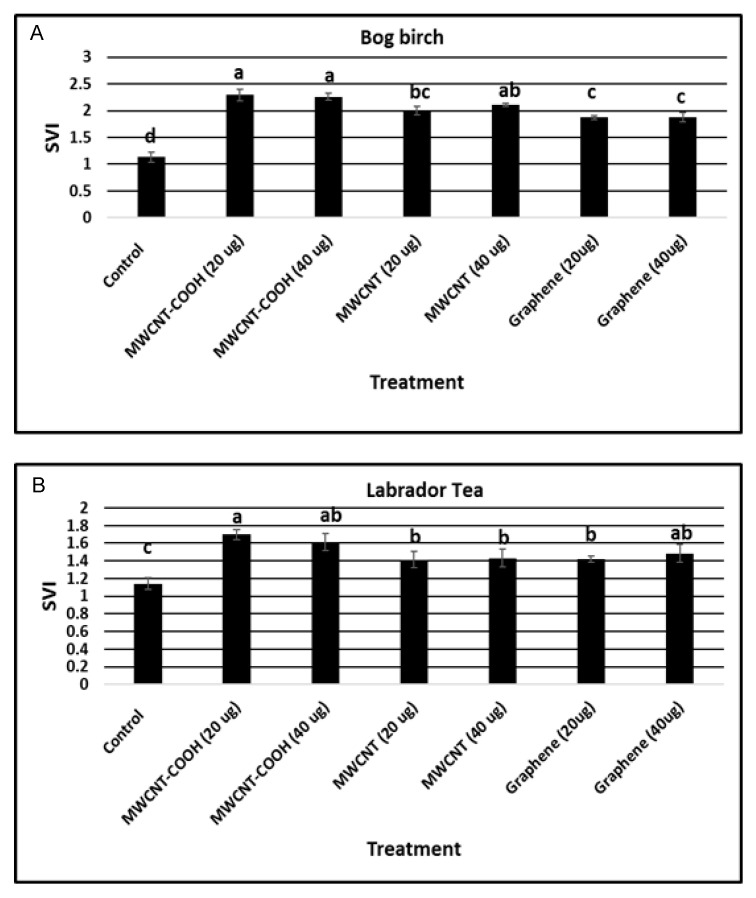
Seedling vigor index of bog birch (**A**) and Labrador tea (**B**). Values in the bar chart represent the means ± standard errors. Means that were significantly different (α = 0.05) are denoted by different letters. Control = no CNTs added. MWCNT–COOH = multiwall carbon nanotubes functionalized with carboxylic acid, MWCNTs = multiwall carbon nanotubes. SVI = seedling vigor index.

Nanopriming significantly (*p* = 0.003) increased the SVI in all experimental treatments (Figure 6A,B). The SVI value (2.4) in bog birch seeds primed with MWCNT–COOH (20 µg/mL and 40 µg/mL) treatments were the highest compared to the control and other treatments. For seeds primed with MWCNTs (20 µg/mL and 40 µg/mL), the SVI value was 2.0 compared to 1.9 for graphene (20 and 40 µg/mL). The SVI value was significantly lower (1.2) in the control compared to all other treatments (Figure 6A). Similar to bog birch, the SVI was significantly higher in Labrador tea seeds primed with CNTs compared to the control (Figure 6B). We observed that MWCNT–COOH at 20 µg/mL resulted in the highest SVI (1.7), while the SVI was similar in value (1.4 to 1.5) when primed with the other two CNPs used (MWCNTs and graphene) at either 20 or 40 µg/mL. The lowest SVI value (1.1) was recorded in the control treatment (Figure 6B).

### 3.6. Possible Role of Lipid Metabolism in Resolving Seed Dormancy in Peatland Boreal Forest Species

**Figure 7:**
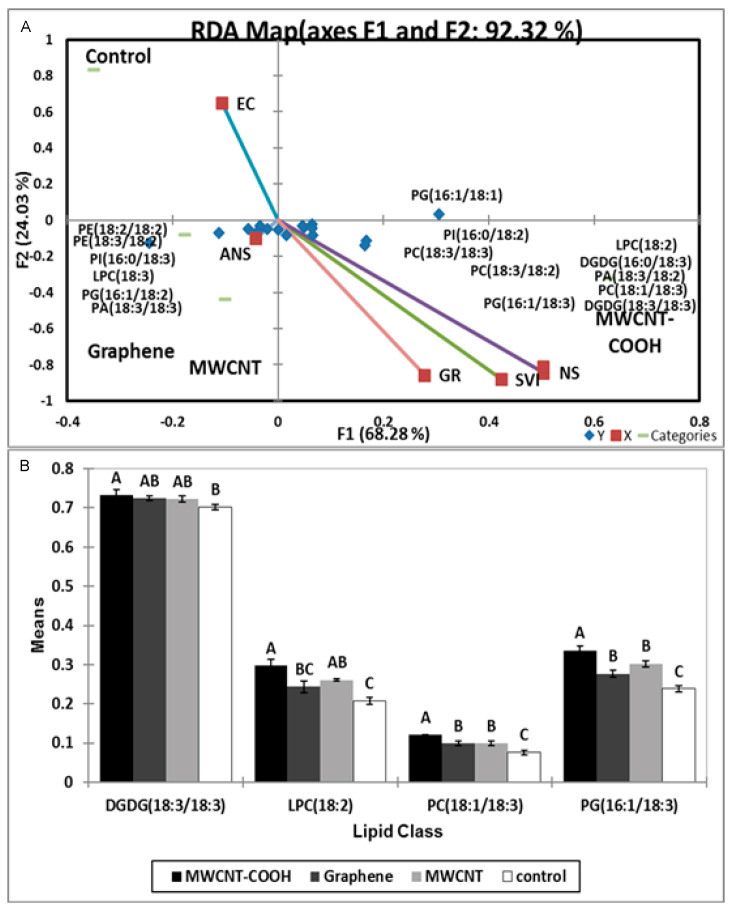
Redundancy analysis (RDA) of Labrador Tea (**A**) for those lipid classes that clustered with MWCNT–COOH treatments. Analysis of variance (ANOVA) (**B**) showed the differences in the segregated lipid molecular species following nanopriming. Values in the bar chart represent the means ± standard errors, n = 100 plants for each treatment. Means that were significantly different (α = 0.05) are denoted by different letters. Control = no CNTs added. MWCNT–COOH = multiwall carbon nanotubes functionalized with carboxylic acid, MWCNTs = multiwall carbon nanotubes. SVI = seedling vigor index, EC = Electrical conductivity, GR = Germination rate, NS = Normal seedlings, ANS = Abnormal seedlings. DGDG = digalactosyldiacylglyceride, LPC = lysophosphatidylcholine, PC = phosphatidylcholine, PG = phosphatidylglycerol, PA = phosphatidic acid, PE = phosphatidylethanolamine.

**Figure 8:**
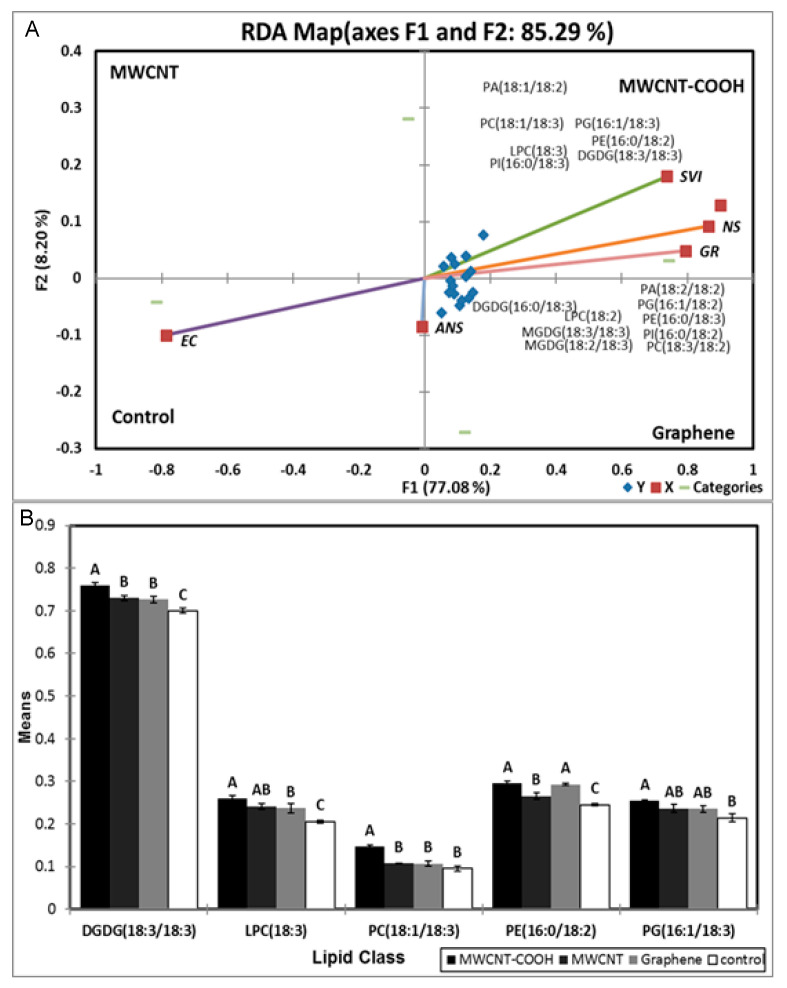
Redundancy analysis (RDA) of bog birch (**A**) for those lipid classes that clustered with MWCNT–COOH treatments. ANOVA analysis (**B**) showed the differences in the segregated lipid molecular species following nanopriming. Values in the bar chart represent the means ± standard errors, n = 100 plants for each treatment. Means that were significantly different (α = 0.05) are denoted by different letters. Control = no CNTs added. MWCNT–COOH = multiwall carbon nanotubes functionalized with carboxylic acid, MWCNT = multiwall carbon nanotubes. SVI = seedling vigor index, EC = Electrical conductivity, GR = Germination rate, NS = Normal seedlings, ANS = Abnormal seedlings. DGDG = digalactosyldiacylglyceride, LPC= lysophosphatidylcholine, PC = phosphatidylcholine, PG = phosphatidylglycerol, PA = phosphatidic acid, PE = phosphatidylethanolamine.

We attempted to evaluate whether lipid metabolism played a role in nanopriming alleviation of seed dormancy and improved seedling vigor in both peatland species. We observed the following classes of membrane lipids were present in the seedlings at germination following nanopriming: cardiolipin (CL), lysophosphatidylcholine (LPC), lysophosphatidylethnolamine (LPE), phosphatidic acid (PA), phosphatidylcholine (PC), phosphatidylehanolamine (PE), phosphatidylglycerol (PG), phosphatidylinositol (PI), phosphatidylserine (PS), digalactosyldiacylglycerol (DGDG), monogalactosyl diglyceride (MGDG), and sulfoquinovosyl diacylglycerols (SQDG) (data not presented). Following redundancy analysis (RDA), most of the physiological parameters (total germination, SVI, or normal seedlings) clustered with the MWCNT–COOH treatment, and this grouping accounted for 92.32 and 85.94% of the total variance for both Labrador tea and bog birch, respectively (Figure 7A and Figure 8A). We observed for Labrador tea that PI (16:0/18:2), PC (18:3/18:3), PC (18:3/18:2), PG (16:1/18:3), LPC (18:2), DGDG (16:0/18:3), PA (18:3/18:2), PC (18:1/18:3), and DGDG (18:3/18:3) were clustered in Q3 with MWCNT–COOH treatment along with germination rate, SVI, and NS (normal seedlings). PE (18:2/18:2), PE (18:3/18:2), PI (16:0/18:3), LPC (18:3), PG (16:1/18:2), and PA (18:3/18:3) were clustered in Q4 with both graphene and MWCNT treatment, along with abnormal seedlings, while EC was clustered with the control (Figure 7A). For bog birch, the PA (18:1/18:2), PC (18:1/18:3), LPC (18:3), PI (16:0/18:30, PG (16:1/18:3), PE (16:0/18:2), and DGDG (18:3/18:3) were clustered in Q2 with MWCNT–COOH along with SVI, germination rate, and normal seedlings, similar to the observation for Labrador tea. On the other hand, DGDG (16:0/18:3), LPC (18:2), MGDG (18:3/18:3), MGDG (18:2/18:3), PA (18:2/18:2), PG (16:1/18:2), PE (16:0/18:3), PI (16:0/18:2), and PC (18:3/18:2) were clustered in Q3 with graphene treatments and abnormal seedlings.

**Figure 9:**
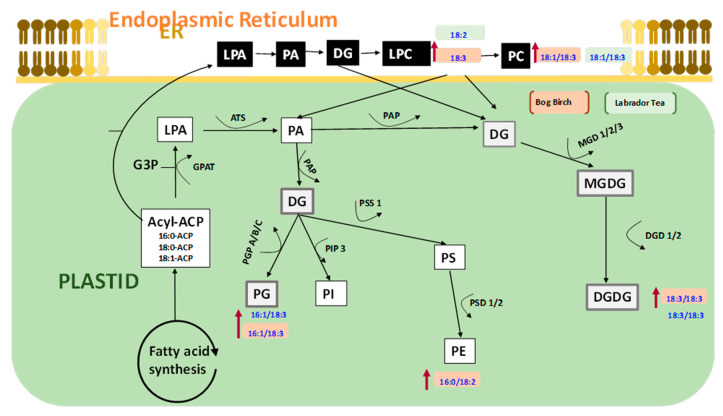
Proposed pathway showing the effects of nanopriming with MWCNT–COOH on the membrane lipid metabolism of peatland boreal forest species in response to possible alleviation of seed dormancy and improved vigor. LPA = Lysophosphatidic, PA = Phosphatidic acid, DG = Diacylglycerol, PC = Phosphatidylcholine, PG = Phosphatidyl glycerol, PI = Phosphatidylinositol, PS = Phosphatidylserine, PE = Phosphatidylehanolamine, MGDG = Monogalactosyldiacylglycerol, DGDG = Digalactosyldiacylglycerol, PGP = Glycerol-3-phosphate phosphatase, EPT = Ethanolamine phosphotransferase, DGD = dialkylglycine decarboxylase, PAP = Phosphatidic acid phosphatase, MGDG = Monoalkylglycine decarboxylase, PSD = Phosphatidylserine decarboxylase, PIP = 1-phosphatidylinositol-4-phosphate 5-kinase.

The EC clustered with the control, and this segregation accounted for 85.29% of the total variability in the data (Figure 8A). Following RDA analysis, ANOVA was employed to examine the effects of the lipid molecular species that clustered with germination rate, SVI, and NS with MWCNT–COOH (the best performing treatment) (Figure 7B and Figure 8B). For Labrador tea, DGDG (18:3/18:3), LPC (18:2), PC (18:1/18:3) and PG (16:1/18:3) were significantly different between the treatments and the control (Figure 9). For PG (16:1/18:3) and PC (18:1/18:3), the means were significantly higher than that in MWCNTs, graphene, and the control. The means of DGDG (18:3/18:3) were higher in the MWCNT–COOH treatment and significantly different from the control, but similar to that of MWCNTs and graphene. In the case of LPC (18:2), seeds primed with MWCNT–COOH was significantly different when compared to the control and graphene (Figure 7B).

For bog birch, DGDG (18:3/18:3) was significantly higher in the nanoprimed seedlings compared to the control, with the highest level recorded in the seedlings primed with MWCNT–COOH (Figure 8B). Similarly, the LPC (18:3) level was elevated in the nanoprimed seedlings compared to the control. Conversely, the PC (18:1/18:3) was significantly lower in the nanoprimed treatments compared with the control. However, PE (16:0/18:2) was observed to be elevated in the nanoprimed seedlings. Though PG (16:1/18:3) was observed to also increase following nanopriming, only in the MWCNT–COOH was the increase significant compared with the control. As such, Pearson’s correlation analysis was conducted among SVI, germination rate (GR) and the significant molecular species (DGDG (18:3/18:3), PC (18:1/18:3), LPC (18:3), and PG (16:1/18:3) to see if there was any association with improvements in germination and seedling vigor following nanopriming. We observed PC (18:1/18:3) and PG (16:1/18:3) were strongly correlated with SVI (r = 0.945, *p* = 0.004; r = 0.933, *p* = 0.007 respectively) (Figure S1). On the other hand, we observed that only PG (16:1/18:3) was significantly (*p* = 0.003) correlated with GR (r = 0.912) (Figure S1). Outputs of the correlation analysis for all the lipid molecular species clustered with MWCNT–COOH, but showed no association with either SVI or GR (Tables S1 and S2).

## 4. Discussion

### 4.1. Effects of Seed Priming with Carbon Nanoparticles on Seedling Quality

In our study, 90 and 95% of bog birch and Labrador tea seeds, respectively, were viable (completely or partially stained) following evaluation using the tetrazolium test (Figure 1A–C). Several studies have demonstrated that tetrazolium test is well suited to determine seed viability. For example, the viability of *Carica papaya* [25], *Nitraria tangutorum*, and *Nitraria sibirica* seed lots [27] were assessed using the tetrazolium test. Consistent with our findings, these authors demonstrated that a 1% tetrazolium solution worked efficiently and quickly in evaluating the viability and quality of these seed lots prior to germination evaluations. Thus, the seeds we used were found to be viable based on the TZ test outputs and the accepted use of this test in the literature to assess seed viability.

Following the tetrazolium test, we also conducted an EC test to further check the viability of the seeds used for our experiments. The higher EC in the control (hydroprimed) compared to the nanoprimed seeds indicated increased ion via cell membrane (control) compared to when seeds were primed with CNTs (Figure 2). These findings indicate the lower membrane potential in nanoprimed seeds. High electrolyte leakage from seed membranes increases the EC and is attributed to lower membrane integrity and seed vigor [28]. Previous reports have also shown that CNT solutions (fullerenes) improved the seed membrane integrity as well as reduced the amount of leached ions from the cell membrane [29]. This is consistent with our findings and support a role for priming seeds with CNTs in improving seed membrane integrity and potential seed vigor during seed germination without compromising the quality of seeds.

The current study showed a higher percentage of normal seedlings following nanopriming compared to the control primed with only water [5]. Only 5% of the seedlings were observed to be abnormal following nanopriming (Figure 3A,B). This finding implies a positive relationship between seed quality and seed priming with CNT. Seed quality is important in seedling production and achieving high-quality seedlings is paramount to plant survival and establishment [30], especially under poor soil and varying environmental conditions. Based on findings in our study, MWCNT–COOH produced the best performance regarding seedling production and quality in both peatland forest species evaluated. Collectively, these results suggest nanopriming dormant peatland boreal forest species with carbon nanoparticles appears to improve seed quality and confirmed that although the seeds were dormant, they were viable prior to germination and seed vigor evaluations.

### 4.2. Effect of Carbon Nanoparticles in Enhancing Germination and Overcoming Seed Dormancy

Bog birch has embryo and seed coat dormancy [11,31]. These dormancies lead to low germination in this species. Poff et al. [32] showed that cold-moist stratification could break embryo dormancy in *Platanthera chapmanii* (terrestrial Orchid) seeds, leading to an 80% increase in germination. Consistent with these findings, we observed that seeds primed with CNTs had higher germination than the controlled specimens, whereas nanopriming combined with cold-moist stratification gave a superior percent and rate of germination compared to when seeds were only stratified (Figure 4A and Figure 5A). Nanopriming with functionalized CNTs has been reported to have superior effects on germination compared to non-functionalized CNTs. For example, Cañas et al. [33] showed that functionalized CNTs were more effective in increasing seed germination rate when compared to non-functionalized CNTs in *Onobrychis arenaria*. Similarly, we observed that MWCNTs functionalized with carboxylic acid (MWCNT–COOH) were superior in breaking both embryo and seed coat dormancies, thereby increasing seed germination in both bog birch and Labrador tea species. The ability of CNTs to penetrate through the seed coat has been proposed as a possible mechanism through which nanoparticles help activate the dormant embryo, resulting in increased seed germination [34]. It must be noted that the exact process of how CNTs can influence this is unclear.

For example, Mondal et al. [35] indicated that CNTs increased the germination and growth of mustard (*Brassica juncea* L.) when MWCNTs were applied at 2.3 μg mL^−1^. The increased germination was attributed to MWCNTs increasing the moisture content and enhancing the water absorption mechanism of root tissues. Conversely, some studies have reported adverse effects of CNPs on seed germination. For example, CNTs in high doses (2000 mg L^−1^) led to phytotoxicity and inhibition of seed germination and root growth in tomato. It is important to note that the tendency of nanoparticles to inhibit germination is more common with metal-based nanoparticles (e.g., Zn and ZnO) than with carbon-based nanoparticles [36]. Bog birch seeds are very tiny and characterized by having a combination of hard seed coats (seed coat dormancy) and embryo dormancies [10]. Both these dormancies lead to reduced seed germination and low seedling vigor. In our study, the treatment of seeds from both peatland species with MWCNT–COOH @ 40 or 20 µg mL^−1^ led to a significant increase in the total number of seeds germinated as well as the germination rate, which is consistent with earlier findings reported for eleven sedge type wetland plant species [37].

This work demonstrates that MWCNT–COOH is superior in breaking embryo and seed coat dormancies in bog birch as well as increased the overall germination in Labrador tea and could be a suitable approach to improve the propagation of these species for forest restoration or reclamation activities following resource mining.

### 4.3. Effect of Carbon Nanoparticles in Enhancing Seedling Vigor Index

According to the Association of Official Seed Analysts (AOSA), seed vigor is an important measure of seed germination potential, which determines the ability for rapid, uniform emergence, and development of normal seedlings under a wide range of field conditions [5]. The seedling vigor index indicates a time-weighted collective germination that quantifies the seedling vigor. Having seeds with high vigor is necessary for effective regeneration. In addition, the potential for unsuitable growth temperatures, water excess or deficiency, high levels of heavy metals or hydrocarbons, and poor nutrients in areas typically revegetated or reclaimed with peatland boreal forest species means that high vigor seeds are needed for successful regeneration [38]. We observed in this study that seeds primed with CNTs had superior vigor, and that the highest vigor was obtained in seeds primed with MWCNT–COOH in both peatland species evaluated (Figure 6). Similar to our findings, silver nanoparticle treated seedlings were reported to attain the greatest growth and about 95% total germination using a concentration of 10–30 µg/mL silver nanoparticle [39]. This enhanced growth in seedlings may be attributed to increased water and nutrient intake by seeds treated with silver nanoparticles. This view is further supported by the work of Srinivasan and Saraswathi [40], which showed that the amount of water intake increased in tomato seeds treated with CNTs, and this contributed to the improved seedling vigor observed. The observation that CNTs, in particular, MWCNT–COOH, was very effective in improving seedling vigor in the two peatland species evaluated is very important regarding reclamation efforts. Seedlings with high vigor are more adaptable to a range of environmental conditions, which in turn improve their chances of growth and establishment at different sites under varying environmental conditions. This is ideal for boreal forest reclamation after resource mining, because often times, the sites to be reclaimed or restored have very harsh environments for plant growth (high salinity, pH, nutrient poor, inadequate, or excess water, etc.). This finding could help in forest regeneration programs because enhanced seedling vigor can be utilized to overcome the harsh conditions present after forests are depleted following anthropogenic disturbances such as oil and gas resource mining.

### 4.4. Possible Role of Lipid Metabolism in Resolving Seed Dormancy in Peatland Boreal Forest Species Following Nanopriming

It has been reported that nanopriming with CNTs have the potential to modify plant cell structure and physiology [16]. In general, membrane lipids are integral to seed germination processes where the seed membrane lipids have been observed to undergo significant metabolism and remodeling as they germinate [41]. This involves a series of processes including a gradual increase of plastidic lipids and membrane lipid reorganization. For instance, Doria et al. [41] reported the changes in seed membrane lipids and membrane reorganization were due to radical mediated oxidative stress during the imbibition process. The levels of phospholipids was shown to be remodeled during germination in soybean seeds, where the level of phosphatidic acid (PA) decreased at the beginning of germination before increasing exponentially during phase I and II of germination, then rapidly declined at the end of germination [42]. This is consistent with the findings in our study, where we observed that the level of different phospholipids changed during the germination of seeds following nanopriming and the resolution of embryo and seed coat dormancy (Figure 7, Figure 8 and Figure 9).

During the seed germination, Elizabeth et al. [43] found that wheat seeds with high vigor were able to quickly accrue membrane lipids, compared to low vigor seeds that did not; this was attributed to the delay in the start of mitosis by low vigor embryos, in contrast to seeds with high vigor. This implies that the high SVI recorded in this research could be attributed to superior bioaccumulation of membrane lipids, as observed in higher levels of DGDG (18:3/18:3), PC (18:1/18:3), PG (16:1/18:3), and LPC (18:1/18:3) in the nano primed seedlings, compared to the control (Figure 8 and Figure 9).

Several phospholipid molecular species were clustered with seed physiological parameters (SVI, GR, NS) when primed with MWCNT–COOH (most effective treatment in improving germination rate, SVI, EC, and normal seedlings). This finding could be attributed to the ability of functionalized multi walled nanoparticles to penetrate the phospholipid bilayer of the cell to cause a change in the structure while acting as a “nanoneedle” in the bilayer [44].

To better understand the connections between membrane phospholipid remodeling and SVI or GR during the resolution of seed dormancy, we conducted a Pearson’s correlation analysis and discovered LPC (18:2) was highly correlated with GR and PC (18:1/18:3), and PG (16:1/18:3) correlated with the SVI (S1). LPC, PC, and PG are vital phospholipids in the cell membrane, and the synthesis or turnover of these phospholipids appears to be associated with improved seed germination and seedling vigor, as shown in our proposed pathway (Figure 9)

In Labrador tea, DGDG (18:3/18:3), LPC (18:2), PC (18:1/18:3), and PG (16:1/18:3) molecular species were elevated following nanopriming, and were highly correlated with improvement in seed vigor and germination (Figure 7 and Figure S1). All these molecular species are connected biosynthetically [45]. PG (16:1/18:3) is synthesized from DG in the presence of the glycerol-3-phosphate phosphatase (PGP) A/B/C enzyme, PC (18:1/18:3) is biosynthesized from DG by the enzyme ethanolamine phosphotransferase (EPT), and PE (16:0/18:2) from PS by the enzyme phosphatidylserine decarboxylases (PSD ½) while DGDG (18:3/18:3) synthesis from MGDG is catalyzed by dialkylglycine decarboxylase (DGD) 1/2 enzyme. PC synthesis occurred in the endoplasmic reticulum of cell, and the other types of lipid are synthesized in the plastid [5,6]. DGDG (18:3/18:3), LPC (18:3), PC (18:1/18:3), PE (16:0/18:2), and PG (16:1/18:3) molecular species were elevated in response to nanopriming with CNTs, particularly by MWCNT–COOH in bog birch. It appears that during the resolution of seed coat and embryo dormancy in bog birch, MWCNT–COOH modulated the accumulation of C18:3 enriched LPC, PG, PE, PC, and DGDG molecular species (Figure 8 and Figure 9).

An overall increase in C18:3 enriched molecular species in response to nanopriming in dormant seeds of both peatland species evaluated implies that the ∆3 desaturase enzyme plays an important role in the modulation of the membrane lipid. In this study, PC (18:1/18:3) and PG (16:1/18:3) were also highly correlated with the seedling vigor index (S1). PC, LPC, PG, DGDG, and PE molecular species enriched with C18:3 fatty acids clustered together with MWCNT–COOH primed seeds and may be associated with the improved dormancy, SVI, and GR observed in this study. This finding indicates that CNTs, in particular MWCNT–COOH, appears to play a role in alleviating seed coat and embryo dormancies through the modulation of C18:3 enriched molecular species, and suggests that these lipid molecular species are highly associated with improved seedling vigor and overall germination in bog birch and Labrador tea important indicator species in peatland ecosystems.

## 5. Conclusions

In this study, we aimed to determine whether selected CNTs could be used as a seed priming agent to break seed dormancies and improve germination or seed vigor in two boreal forest peatland species and if altered cell membrane lipid metabolism might be associated with the improved germination and seedling vigor in the tested species. We showed for the first time that CNTs could break seed dormancy and help increase germination and seedling vigor in two boreal forest species common in peatland ecosystems. The study demonstrated that nanopriming of dormant seeds of two boreal peatland species enhanced seed germination and the seedling vigor index, and that the improved germination and seedling vigor were highly correlated with the levels of C18:3 enriched PC (18:1/18:3), PG (16:1/18:3), and LPC (18:1/18:3) molecular species. Of all the treatments in the experiment, stratification combined with MWCNT–COOH nanopriming has proven to be the most effective in overcoming seed dormancy, aiding germination (rate, total germination and normal seedlings), and improving seedling vigor. Nanoprimed seeds with MWCNT–COOH appears to have potential in improving the seed germination and seedling vigor of boreal forest species with morphological and physical seed dormancy issues, which has great importance in overcoming the challenges associated with mass propagation of these native non resource boreal peatland species for forest reclamation, revegetation, or regeneration activities following anthropogenic disturbances such as oil and gas resource mining.

## Supplementary Materials

The following are available online at https://www.mdpi.com/2079-4991/10/9/1852/s1, Figure S1: Correlations between lipid molecular species, seedling vigor index (A,B) and seed germination (C). Correlation done for only R1, R2, and R3 of MWCNT–COOH treatments of both bog birch and Labrador tea and only means are presented. R = Pearson’s correlation coefficient, *p* = alpha at 0.05. n = 100 plants per treatment. SVI = Seedling vigor index, GR = germination rate, PG = phosphatidyl glycerol, PC = phosphatidylcholine. Table S1: Lipid molecular species clustered in the same quadrants as MWCNT–COOH, SVI, and GR following RDA analysis, but are not significantly correlated following Pearson correlation analysis. N = 100 plants per treatment. Table S2: Lipid molecular species clustered in the same quadrants as MWCNT–COOH, SVI, and GR following RDA analysis, but were not significantly correlated following the Pearson correlation analysis. N = 100 plants per treatment.
