# Supplementary material for: Carbon Nanotubes Improved the Germination and Vigor of Plant Species from Peatland Ecosystem Via Remodeling the Membrane Lipidome
Source: Nanomaterials (Basel). 2020 Sep 16;10(9):1852. doi: 10.3390/nano10091852 (PMC7557389; doi:10.3390/nano10091852)
Supplement: Supplementary file 1 [file nanomaterials-10-01852-s001.pdf]

nanomaterials

Supplementary Materials

Carbon Nanotubes Improved the Germination and Vigor of Plant Species from Peatland Ecosystem Via Remodeling the Membrane Lipidome

Md. Hossen Ali [1], Jean-Marie Sobze [2], Thu Huong Pham [1], Muhammad Nadeem [1,*], Chen Liu [1], Lakshman Galagedara [1], Mumtaz Cheema [1] and Raymond Thomas [1,*]

[1] School of Science and the Environment/Boreal Ecosystem Research Facility, Grenfell Campus, Memorial University of Newfoundland, Corner Brook, NL A2H 5G5, Canada; mhali@grenfell.mun.ca (M.H.A.); tpham@grenfell.mun.ca (T.H.P.); cliu@grenfell.mun.ca (C.L.); lgalagedara@grenfell.mun.ca (L.G.); mcheema@grenfell.mun.ca (M.C.)

[2] Northern Alberta Institute of Technology, Boreal Research Institute, 8102-99 Avenue, Peace River, AB T8S 1R2, Canada; jeanmars@nait.ca

* Correspondence: muha.nadeem@gmail.com (M.N.); rthomas@grenfell.mun.ca (R.T.)

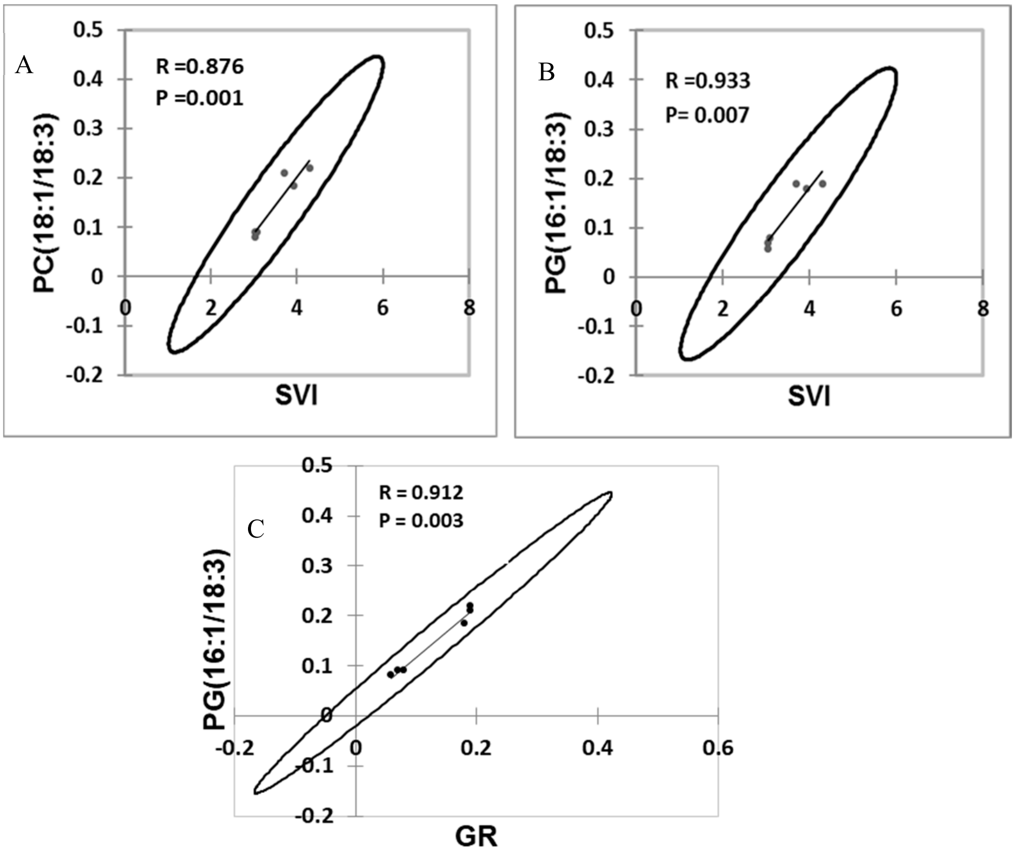

Figure S1. Correlations between lipid molecular species, seedling vigor index (**A**,**B**) and seed germination (**C**). Correlation done for only R1, R2, and R3 of MWCNT-COOH treatments of both bog birch and Labrador tea and only means are presented. R = Pearson's correlation coefficient, p = alpha at 0.05. n = 100 plants per treatment. SVI = Seedling vigor index, GR = germination rate, PG = phosphatidyl glycerol, PC = phosphatidylcholine.

Table S1. Lipid molecular species clustered in the same quadrants as MWCNT-COOH, SVI and GR following RDA analysis, but are not significantly correlated following Pearson correlation analysis. N = 100 plants per treatment.

Labrador tea	
**Name of lipid Classes**	**Level of significance**
PI (16:0/18:2)	NS
PC(18:3/18:3)	NS
PC(18:3/18:2)	NS
PA(18:3/18:2)	NS
DGDG(16:0/18:3)	NS
LPC (18:2)	NS
DGDG(18:3/18:3)	NS

Table S2. Lipid molecular species clustered in the same quadrants as MWCNT-COOH, SVI and GR following RDA analysis, but are not significantly correlated following Pearson correlation analysis. N = 100 plants per treatment.

Bog birch	
**Name of lipid Classes**	**Level of significance**
PI (16:0/18:3)	NS
PA(18:1/18:2)	NS
LPC (18:3)	NS
PE (16:0/18:2)	NS
DGDG(18:3/18:3)	NS
